# Modern Health Service Utilization and Associated Factors among Adults in Southern Ethiopia

**DOI:** 10.1155/2021/8835780

**Published:** 2021-01-11

**Authors:** Shimelash Bitew Workie, Niguse Mekonen, Mulugeta W. Michael, Getahun Molla, Solomon Abrha, Zewde Zema, Takele Tadesse

**Affiliations:** ^1^School of Public Health, College of Medicine and Health Sciences, Wolaita Sodo University, Wolaita Sodo, Ethiopia; ^2^School of Medicine, College of Medicine and Health Sciences, Wolaita Sodo University, Wolaita Sodo, Ethiopia; ^3^School of Pharmacy, College of Medicine and Health Sciences, Wolaita Sodo University, Wolaita Sodo, Ethiopia

## Abstract

**Background:**

The Ethiopian government is striving to improve the health status of its population through the expansion and strengthening of primary health care services in both rural and urban settings of the country. The study aimed to measure modern health service utilization and associated factors in Wolaita Sodo town, Ethiopia.

**Method:**

A cross-sectional study design was implemented from May to June 2019 in Wolaita Sodo town, Ethiopia. All 786 study participants were selected by multistage systematic random sampling. Data were collected by face-to-face interviews using a pretested structured questionnaire. Data were collected by an open data kit. Stata window version 15.0 was also employed for statistical analysis. Multiple logistic regression was conducted, and a 95% confidence interval was considered for interpretation.

**Result:**

Health service utilization was 77.2% with (95% CI of 74.1%, 80.0%). In terms of health facilities in which they visit, first 50.6% were at the public health center and 25.5% of them were at Teaching and Referral Hospital. Respondents with marital status married and widowed have higher odds of utilizing health services compared to single marital status (AOR: 2.96; 95% CI: 1.7–5.2 and 9.0; 95% CI: 1.69–48.0), respectively. Respondents with middle and highest wealth status have higher odds of health service utilization than poor wealth status with AOR (1.75 95% CI 1.03–2.97 and 1.58 95% CI; 1.01, 2.77). Similarly, respondents who had chronic disease and perceived poor health status have higher odds of health service utilization.

**Conclusion:**

Modern health services utilization was found to be unsatisfactory. Being married, wealth status being middle and high, having chronic health conditions, and having poor perceived health status were found to have a statistically significant association with health service utilization.

## 1. Introduction

The 2030 Sustainable Development Goals (SDG) emphasize having all people receive the quality health services they need without financial hardship. Critical to attaining universal health coverage (UHC) is a formal monitoring mechanism to assess progress. The UHC service coverage index, measuring progress on SDG indicator 3.8.1, increased from a global average of 45% in 2000 to 66% in 2017 [1].

The health service utilization rate in Africa is low and sub-Saharan Africa, in particular, is very low, ranging from only 0.2 annual visits to 2 visits [2].

The utilization of health care services is an important public health and policy issue in developing countries for the achievement of universal health coverage [3]. Access to and utilization of health services are key to the improvement of health outcomes in low- and middle-income countries (LMICs) [4]. In recent years, the Ethiopian government is investing lots of money to improve the health care of its nation. This is considered one of the top lists of transformational agendas by the government [5]. According to the Ministry of Health, due emphasis is given to the improvement of health care services towards the expansion and strengthening of primary health care emphasizing maternal and child health and prevention of both communicable and noncommunicable diseases. Hence the government believes that the delivery of quality health services at all levels of the stages of the health system is central to improving the health status of the population. Improving the quality of health services implies the improvement of supply and demand-side interventions and the health and health-related regulatory aspect [6].

Ethiopia is one of the sub-Saharan countries most affected by the high disease burden reflected by the high rates of maternal and child mortality. Based on 2015 point estimates, noncommunicable diseases were the leading contributor to age-standardized death rates in Ethiopia, causing 710.9 (468.8–1036.2) deaths per 100,000; however, communicable, maternal, neonatal, and nutritional (CMNN) diseases were the leading causes of premature mortality, with a rate of 17,950.6 (14,377.9–22,768.8) per 100,000. CMNN diseases caused more deaths in young people, those between 15 and 49, than other causes in 2015 [7].

Health service utilization is one of the key elements for alleviating morbidity and mortality. In Ethiopia, different studies tried to estimate health service utilization. Therefore, the present study aims to measure the utilization of health service of adults and associated factors in Wolaita Sodo town, Ethiopia, and to suggest ways to improve and strengthen health centers to improve quality of care and reduce the burden on university hospitals.

## 2. Methods

### 2.1. Study Area

The study was conducted in Wolaita Sodo town which is the capital city of Wolaita Zone. The town is southwest 327 km far from Addis Ababa and 166 km South from the regional Capital city, Hawassa. The town is located at an altitude of 1500–2500 m above sea level with an area of 82.1 km^2^. The town has three subtowns and 11 administrative Kebele. The estimated total population number is 143,438 with 70285 males, 73153 females. The town has 14,551 HH, one referral hospital, one private hospital, 11 health posts, 3 health centers, and 24 private health institutions.

### 2.2. Study Design and Period

Community-based cross-sectional study design was implemented from May to June 2019.

#### 2.2.1. Source Population

All adults in Wolaita Sodo town were the source population. All selected adults were our study population. The sampling unit were households.

#### 2.2.2. Inclusion and Exclusion Criteria

All adults greater than 18 years and who lived more than 6 months in the town were included. Severely sick adults were excluded from the study.

#### 2.2.3. Sample Size Determination and Sampling Strategy

The total sample size 786 was calculated using single population formula and assuming the proportion of modern health services utilization of 41.8% [8], 95% confidence level, and 5% margin of error and finally adding 5% nonresponse rate. Multistage systematic random sampling was used. The first 4 kebeles were selected out of 11 kebeles. Then every k^th^ household based on their specific house numbers was selected. The head of the family or elder of the family members was interviewed for the questionnaire.

#### 2.2.4. Data Collection Procedures and Data Quality

Data were collected using a pretested, interviewer-administered questionnaire adapted from the World Health Organization Manual for the Household Survey to Measure Access and Use of Medicines. The questionnaire was initially prepared in English and translated to Amharic and back to English to maintain consistency of the language. Finally, the Amharic version was used for data collection. Data collection was facilitated by the Open Data Kit (ODK). Trained interviewers who were trained for this purpose were to administer the questionnaire.

### 2.3. Variables

#### 2.3.1. Dependent Variable

Health Service utilization.

#### 2.3.2. Independent Variables

Sociodemographic and behavioral factors were age, sex, marital status, occupation, educational status, and perceived health status.

### 2.4. Operational Definitions of Terms

#### 2.4.1. Modern Health Services

Modern health services in this study include public and private licensed health institutions (hospitals, health centers, clinics, and private not-for-profit organizations).

#### 2.4.2. Modern Health Services Utilization

Health services utilization in the study refer to a measure of the health of the population whether the respondent went to modern health institutions in the last 12 months before the study. It is a dichotomous variable based on the survey question ^a^Did you go for health care in the last 12 months?º Yes = 1 and No = 0.

### 2.5. Data Quality Measures

The quality of data was ensured by pretesting the data collection tools on 5% of the participants in Bodity town, which has similar sociodemographic characteristics with Woliata Sodo town. Data were collected by 6 BSc in health science students and 3 Masters of Public Health (MPH) graduates supervised the process. Overall Supervision was also carried out at the spot by the principal investigator. The training was given to both data collectors and supervisors on the questionnaire, content interviewing technique, the purpose of the study, and how to maintain the confidentiality of information. To maximize the quality, data were collected by mobile tables. The collected data were checked for completeness and clarity by assigned supervisors and principal investigators.

### 2.6. Data Management and Analysis

Data were collected by mobile tablets using Open Data Kit (ODK). ODK is a free mobile application that is designed by Excel. Then data were exported to Excel to Stata Version 15. Descriptive analysis was done for sociodemographic variables using frequency tables and measures of central tendency. Wealth index (WI) was computed by using a principal component factor/PCF/analysis.

To assess the presence of an association between dependent and independent variables, OR with 95% confidence intervals (95% CIs) and the *p* value were reported on all proportions. The binary logistic regression was used to identify the variables associated with health service utilization. Those of the *p* values less than 0.25 were the candidate variable for multiple logistic regression. In multiple logistic regression analysis, those variables with a *p* value < 0.05 and 95% CI were considered as significant determinates of health service utilization.

Multicollinearity was checked by using the Variance inflation factors/VIF/and Tolerance test. The Hosmer-Lemeshow tests were checked to assess model goodness-of-fit.

## 3. Result

### 3.1. Sociodemographic Characteristics

The total number of participants was 768, which makes the response rate 97.7%. Of the total respondents, 73% were females. Seventy-one percent were married. Sixty-four percent of the respondents were Protestant by religion. The median age was 31 years with a range of 18–60 years, and 23.2% were in the age category 34–42 years. Most of the study participant's occupations were housewives 193 (25.1%). Fifty-one percent of the respondents were middle wealth status (Table 1).

Almost all 97.5% had a health facility nearby with a mean of 30-minute walking distance from health facility ranging from 3 minutes to 60 minutes. Perceived health status was rated to be good for 74.7%. Eleven percent of the respondents have at least one chronic disease. Ninety-eight percent of the respondents have visited health facilities at least once in their lifetime (Table 2).

### 3.2. Health Service Utilization

Health service utilization was 77.2% of the respondents with 95% CI of 74.1%, 80.0% the last year. Thirty-three percent of respondents visited once in the last 12 months. Almost forty percent of the respondents seek health care for cold medical conditions (OPD). Interims of the facility in which they visit first 50.6% were at public health centers and 25.5% of them were at teaching and referral hospitals (TRH). Of those who were visiting teaching and referral hospitals, 90.07% were self-referral. Fifty-one percent of the respondents were satisfied with the service they get (Table 3).

### 3.3. Health Service Utilization-Associated Factors

Multiple logistic regression analysis showed that respondents with marital status married and widowed have higher odds of utilizing health services compared to single marital status (AOR: 2.96; 95% CI: 1.7–5.2 and 9.0; 95% CI: 1.69–48.0), respectively. Respondents with middle and highest wealth status have higher odds of health service utilization than poor wealth status with AOR (1.75 95% CI 1.03–2.97 and 1.58 95% CI; 1.01, 2.77), respectively. Similarly, respondents who had chronic disease have higher odds of health service utilization with AOR: 7.8, 95% CI: 2.3–26.5, than their counterparts. Those with perceived poor health status were 96% more likely to utilize health services than their counterparts (Table 4).

## 4. Discussion

Health service utilization was 77.2% (95% CI of 74.1%, 80.0%) in Wolaita Sodo town. This finding was higher than in Northeast Ethiopia, which is 41.8% [8]. It is also higher than the study done in Jima, which was 45.6% [9]. This study might only be for urban residents because urban residents have access to health facilities. It is comparable to a study done in Kenya [10]. Half of them were first to visit primarily at the public health center and 25.5% of them were at teaching and referral hospitals. Of those who visited university teaching and referral hospitals, 90.07% were self-referral.

Factors that affect health service utilization were marital status, wealth status, presence of chronic disease, and perceived health status.

Marital status being married and divorced were more likely to utilize health service. This finding is similar to a study done in Jimma [9]. Wealth status was the main factor in the utilization of modern health services. Middle and high wealth status were more utilized to health services compared to the poor. This finding was similar to the study done by Dessie [8] and Jimma [9] in Ethiopia. Poor people have lower coverage even for basic services such as immunization, sanitation, and antenatal care [1]. This is also in line with the study done in Ghana [11]. This might be due to user fees related to the health service. Removing user fees increases the utilization of curative healthcare services, usually in the form of one sharp step-up following fee removal. Those high and middle wealth status people are capable of paying more than the poor [12].

Those with chronic diseases were 7.8 more likely to use modern health services. This is in line with previous studies, which reported that having chronic conditions was a strong predictor of health care utilization [8, 9]. This is also in line with studies done in Ghana, which reported that having multiple chronic conditions is a predictor of health service utilization [11].

Perceived health status was also found to be associated with the utilization of health services. Respondents who perceived that their health status was poor were more likely to visit the health institution. This is consistent with the study done in Dessie [8]. This might be because those who perceived that their health was in poor condition seeks treatment and would visit the health institution.

This study had some limitations.

## 5. Conclusion and Recommendation

Modern health services utilization was found to be unsatisfactory. Being married on marital status, wealth status being middle and high, having chronic health conditions, and having poor perceived health status were found to have a statistically significant association with health service utilization. This indicates that improving the wealth status of the population will possibly result in better utilization of health care.

## Figures and Tables

**Table 1:** Sociodemographic characteristics of Wolaita Sodo residents attending health care utilization survey at Wolaita zone, July 2019 (*N* = 768).

Variable		Frequency (768)	Percentage
Sex	Female	563	73.3
	Male	205	26.7

Age category	18–23	168	21.3
	23–28	146	19.1
	28–34	157	20.4
	34–42	178	23.2
	≥42	158	20.57

Religion	Protestant	494	64.3
	Orthodox	214	27.9
	Catholic	10	1.3
	Muslim	38	5.0
	Joba witness	5	0.6
	Seventh-day adventist	5	0.6
	Others	2	0.3

Marital status	Married	550	71.6
	Single	160	20.8
	Widowed	35	4.6
	Divorce	23	3.0

Educational status	Unable to read and write	51	6.6
	Can read and write	46	6.0
	Primary education [1–8]	167	21.6
	Secondary education [9–12]	264	34.2
	College/university	245	31.7

Spouse's educational	Unable to read and write	51	6.6
	Can read and write	46	6.0
	Primary education [1–8]	167	21.7
	Secondary education [9–12]	261	21.7
	College/university	243	31.6

Main occupation	Housewife	193	25.1
	Student	99	12.9
	Employee (gov, private, NGO)	161	21.0
	Merchant	175	22.8
	Daily laborer	45	5.9
	No job	72	9.4
	Others^*∗*^	23	3.1

Wealth status	Poor	109	14.19
	Middle	392	51.04
	Highest	267	34.77

^*∗*^Pension, carpenter, driver, evangelical, and assistant driver.

**Table 2:** Health facility access and perceived health status of Wolaita Sodo residents attending health care utilization survey at Wolaita zone, July 2019 (*N* = 768).

Variable		Frequency (768)	Percentage
Public health center near to your locality	Yes	749	97.5
	No	19	2.5

The distance of health service in walking minutes	Less than 30 minutes	340	44.3
	Greater than and equal to 30 minutes	428	55.7

Health status	Poor	194	25.3
	Good	574	74.7

Chronic disease	No	679	88.4
	Yes	89	11.6

Ever visited facilities	Yes	756	98.4
	No	12	1.6

**Table 3:** Health service utilization of Wolaita Sodo residents attending health care utilization survey at Wolaita zone, July 2019 (*N* = 768).

Variable		Frequency (768)	Percentage
Visited last year	Yes	593	77.2
	No	175	22.8

Number of times per year visited (593)	Once	201	33.9
	Twice	130	21.92
	Three times	75	12.65
	Many	171	28.84
	Do not know	16	2.7

Reason for health utilization (593)	For child health	98	16.12
	For delivery	20	3.29
	For general health checkup	28	4.61
	For cold medical conditions (OPD)	241	39.64
	Maternal and child health (MCH)	88	14.47
	Others	23	3.78
	Chronic diseases follow-up	70	11.51
	For emergency condition	23	3.78

Type of facility utilized (593)	Nongovernmental organizations (NGO)	2	0.34
	Private clinic/hospital	139	23.48
	Public health center	300	50.68
	Wolaita Sodo University Teaching and Referral Hospital	151	25.51

Source of referral for TRH (151)	Referred from another facility	15	9.93
	Self-referral	136	90.07

Satisfaction rate for utilized (593)	Very dissatisfied	17	2.87
	Dissatisfied	86	14.5
	Satisfied	305	51.43
	Very satisfied	185	31.2

**Table 4:** Factors associated with health care utilization of Wolaita Sodo residents, July 2019 (*N* = 768).

Variables (*n* = 768)	Category	Health service utilization		*p* value	COR (95% CI)	AOR (95% CI)
		Yes (%)	No (%)			
Sex	Female	440 (78.2)	123 (21.8)	0.30	1	
	Male	153 (74.6)	52 (25.4)		0.82 (0.57, 1.2)	

Age category	≤23	81 (62.8)	48 (37.2)		1	
	23–28	108 (74.0)	38 (26.0)	0.047	1.7 (1.01, 2.8)	
	28–34	127 (80.9)	30 (19.1)	0.001	2.5 (1.5, 4.3)	
	34–42	142 (79.8)	36 (20.2)	0.001	2.3 (1.4, 3.9)	
	≥42	135 (85.4)	23 (14.6)	<0.001	3.5 (2.0, 6.1)	

Marital status	Single	95 (59.4)	65 (40.6)		1	1
	Married	454 (82.6)	96 (17.4)	<0.001	3.2 (2.2, 4.7)	2.69 (1.7, 5.2)
	Widowed	33 (94.3)	2 (5.7)	0.001	11.3 (2.6, 48.7)	9.0 (1.7, 48.0)
	Divorced	11 (47.8)	12 (52.2)	0.297	0.6 (0.26, 1.5)	0.5 (0.2, 1.5)

Educational status	Cannot read and write	43 (84.3)	8 (15.7)		1	
	Can read and write	41 (89.1)	5 (10.9)	0.489	1.5 (0.5, 5.5)	
	Primary education [1–8]	115 (68.9)	52 (31.1)	0.034	0.4 (0.18, 0.93)	
	Secondary education [9–12]	189 (72.4)	72 (27.6)	0.080	0.8 (0.22, 1.1)	
	College/TVET/university	205 (84.4)	38 (15.6)	0.993	1.6 (0.4, 2.3)	

Main occupation	Daily laborer	35 (77.8)	10 (22.2)		1	
	Employee (gov, private, NGO)	132 (82.0)	29 (18.0)	0.52	1.3 (0.6, 2.9)	
	Housewife	152 (78.8)	41 (21.2)	0.88	1.1 (0.5, 2.3)	
	Merchant	137 (78.3)	38 (21.7)	0.941	1.0 (0.5, 2.3)	
	No job	55 (76.4)	17 (23.6)	0.862	0.9 (0.4, 2.2)	
	Student	65 (65.7)	34 (34.3)	0.146	0.5 (0.2, 1.2)	
	Others	17 (73.9)	6 (26.1)	0.722	0.8 (0.3, 2.5)	

Wealth status	Poor	75 (12.7)	34 (19.4)		1	1
	Middle	304 (51.2)	88 (50.3)	0.061	1.5 (1.0, 2.5)	1.75 (1.03, 2.97)
	Highest	214 (36.1)	53 (30.3)	0.019	1.8 (1.1, 3.0)	1.58 (1.01, 2.77)

Chronic disease	No	507 (74.7)	172 (25.3)	<0.001	1	1
	Yes	86 (96.6)	3 (3.4)		9.7 (3.0, 31.1)	7.8 (2.3, 26.5)

Distance by walking minutes	Less than 30 minutes	259 (76.2)	81 (23.8)	0.541	1	
	Greater than and equal to 30 minutes	334 (78.0)	94 (22.0)		1.1 (0.8, 1.6)	

Health status	Poor	168 (28.3)	26 (14.8)	<0.001	2.26 (1.43, 3.56)	1.96 (1.19, 3.23)
	Good	425 (71.7)	149 (85.2)		1	1

AOR = adjusted odds ratio, COR = crude odds ratio, ^*∗*^≤0.05, ^*∗∗*^≤0.01.

## Data Availability

The datasets analyzed during the current study are available from the corresponding author upon reasonable request.

## Abbreviations

AOR: Adjusted odds ratio
CI: Confidence interval
CMNN: Communicable, maternal, neonatal, and nutritional
COR: Crude odds ratio
LMICs: Low- and middle-income countries
ODK: Open data kit
OPD: Outpatient department
TRH: Teaching and referral hospital
SDG: Sustainable development goals
UHC: Universal health coverage
WI: Wealth index.
